# A novel unit value method for urban ecosystem services assessment

**DOI:** 10.1371/journal.pone.0324185

**Published:** 2025-05-21

**Authors:** Yunhua Lin, Xudong Jia, Donghoon Lee, Ziwei Zhou

**Affiliations:** 1 Department of Architectural and Planning, Hubei University of Technology, Wuhan, Hubei, China; 2 College of Engineering and Computer Science, California State University, Northridge, Los Angele, United States of America; 3 Department of Architecture, Seoul National University of Science and Technology, Seoul, Korea; 4 Department of marketing, College of Economics and Management, Huazhong Agricultural University, Wuhan, Hubei, China; Sohar University, OMAN

## Abstract

Land use and land cover in cities experience complicated changes in response to rapid urbanization, requiring significant impact on ecosystem service value. However, traditional valuation methods tend to ignore artificial ecosystem. This study develops a novel Unit Value (UV) method to evaluate urban ecosystem service values with both natural and artificial ecosystems considered comprehensively. This method is used to reveal the spatial-temporal evolution of ESV in Wuhan from 1996 to 2018. Additionally, Pearson’s correlation between ESVs and driving factors is studied by using a multiple regression model. The results show that: (1) Ecosystem service values are observed to increase by 20.94% because of land use and land cover increases in woodland ((32.21%) and man-made wetland (61.73%) in 1996–2018, respectively. (2) ESVs declined in central districts and increased in suburban districts, with a clear imbalance between “high central areas” and “low suburban areas”. (3) Human activities play a more important role in urban ecosystem compared to natural environmental factors, especially agriculture, per capita GDP, and population. This study provides a distinctive method for the spatiotemporal evaluation of urban ecosystem service values, establishing an insightful basis for urban sustainable development.

## Introduction

Ecosystem services (ESs) refer to the direct or indirect benefits obtained by humans through the structure, processes, and functions of ecosystems [1]. Ecosystem service value (ESV) can be quantified in monetary terms as the economic value of ecosystem services [2]. As global urbanization rate exceeds 50% [2], more than two-thirds of the world population are expected to live in cities by 2050 [3], with nearly 90% of the new population in Asia and Africa, especially in China and India [4]. However, accompanied by the rapid urbanization process, land use and land cover (LULC) changes accelerate and natural ecosystems transform into artificial ecosystems[5,6], resulting in changes in ESs such as biodiversity, climate change, and hydrological regulation [2,7]. Therefore, it is of great significance to explore a method for evaluating ESV of urban ecosystems to determine optimized allocation of resources and environment.

Many researchers have studied ESs [6–11]. A series of methods have been applied in quantitative evaluation of urban ESs, including market-value methods [12], value transfer methods [13], and investment models [11]. These methods have provided criteria and indicators for evaluating ESVs at various levels. However, these methods used natural ecosystems as cases to evaluate ESVs of urban ecosystems and neglected impacts of artificial ecosystems on urban ESVs. Additionally, these studies have less attention on ecosystem services’ spatiotemporal heterogeneity, thus reducing the accuracy of urban ESV assessment and restraining ES applications in ecological protection policy formulation. Therefore, it is necessary to explore the following issues: How to evaluate the service value of artificial ecosystems? How to consider temporal dynamics and spatial heterogeneity of ESV assessment, and how to improve the accuracy of urban ecosystem service value assessment?

In addressing the above issues, our study aims to develop a new method, assess urban ESVs of natural, semi natural, and artificial ecological subsystems, and analyze impacts of land-use/land over on urban ESs. This paper extends the existing literature and has the following key contributions:

- A new ESV assessment method is developed to consider artificial ecosystems. Equivalent factors for built-up land and man-made wetland plus other equivalent factors for natural ecosystem are integrated to form a foundation for urban ESV assessment. The new method is advantageous over previous ESV methods and is proven to be applicable for ESV assessment of districts, cities, regions, or provinces in China.
- A multidimensional spatiotemporal adjustment approach is developed for composite ecosystems. It integrates natural, economic, and spatial characteristics to adjust equivalent factors. Theoretically this approach overcomes the limitations of traditional ESV assessments by providing a new equivalent factor table suitable for urban ecosystems. Moreover, it can assess ESV variations and distinguishes positive and negative driving forces, thus providing theoretical foundations and practical guidance for sustainable urban development.

The remainder of this paper is as follows: The Literature review section provides the literature review related to this study. The Study area section delineates the study area. The Methodology section introduces the whole methodology including our database and analytical methods. The Results section unfolds the research results, underpinned by unit value method (UV model) and ordinary least squares (OLS model). The Discussions section discusses the empirical outcomes. The Conclusions and future work section summarizes conclusions, states the limitations and offer suggestion of future works.

## Literature review

Ecological services are products and services produced directly or indirectly through processes and functions of ecosystems [8,14,15]. Monetary value assessment of ecosystem services helps us understand benefits provided by ecosystems [16]. It therefore is important to urban and regional land use planning [6,16]. Two groups of approaches have been developed for assessing ESVs (see Table 1). The first group consists of data-driven methods in which evaluating ESVs involves 1) biomass quality assessment of ecosystem services using ecological models/ecological production functions (e.g., microclimate regulation models, air quality regulation models, water purification model, etc.) and 2) functional monetary assessment of ecosystem services using economic valuation techniques (e.g., market price, replacement cost, contingent valuation, etc.).[17–21] Many data-driven approaches assume that each ecosystem service can be assessed by an ecological physical model in which input parameters are employed to simulate an evolving ecological process. Data-driven methods typically are aimed at a single ecosystem[22] and are applied to estimate ESVs at a small spatial scale[23]. As spatial and temporal scales increase in ecological physical models, researchers have taken advantages of machine learning technologies to explore complex ecosystems and assess interactions between environments and human activities[24]. A significant set of machine learning algorithms have been developed[25–28]. Using trained algorithms, researchers have started estimating ecosystem service values. Although data-driven methods are promising with the support of machine learning technologies, large-scale machine learning models (like GPT 4.0 for supporting the ChatGPT platform) have not been developed yet for ESVs assessment and predictions.

**Table 1:** Methodologies adopted for ESV valuation. The classification of assessment methods is based on [8,10,26–29].

Type	Sub-type	Methodology	Specific method	Assessed Ess	Pros	Cons	Reference	Suggestion
**The data-driven methods**	Biomass quality assessment	Biophysical modeling	Heat flux equation	Microclimate regulation	They have strong spatial matching as they can simulate ecological processes	complex calculations, numerous parameters and Inconsistent models	[26–29]	This paper suggests applying the methods to a single ES and at a small spatial scale

			Dry deposition model, etc.	Air quality regulation				
			SWAT, etc.	Soil erosion and flood prevention				
		ESV model	ARIES model	Cultural and regulation services	It uses integrated code and can modify conditions			
			SolVES model	Cultural services	Strong spatial explicit effect			
			InVEST model	Multiple services	Input data criteria, without any expertise	It is very sensitive to data changes		
		Energy valuation	Energy valuation	Multiple services	Truly reflecting ecosystems	complex calculations and data limits		
	Functional monetary assessment	Direct market evaluation	Market price method	Provision services	Real market transaction prices	Underestimate ESVs (no non use value)		
			Replacement cost	Regulation services				Misestimate ESVs (data limits)
		Alternative market evaluation	Travel cost method, etc.	Cultural services	Easy to implement.	Underestimate ESVs (only considers transportation costs		
		Simulated market evaluation	Contingent valuation, etc.	Multiple services	It can evaluate non use value	Controversy on hypothesis and respondent bias		
**The value-driven methods**	Value transfer method		Basic value transfer	Multiple services	Less data, simple operation	Neglecting specific situations of each country, artificial ecological communities and spatial heterogeneity	[8]	When appled for multiple ESs and large scales in China, it is recommended to consider local adjustment factors, artificial ecosystems and spatiotemporal heterogeneity
			Expert modified value transfer	Multiple services	Less data, simple operation, Adjusts values for local ecosystem conditions	Neglecting artificial ecological communities and spatial heterogeneity	[10]	**When appled for multiple ESs and large scales in China, it is recommended to consider artificial ecosystems and spatiotemporal heterogeneity**

The second group of approaches contains unit value-based methods (later referred to as UV methods) (e.g., basic value transfer, expert modified value transfer). These methods, originally proposed by Costanza et al. in 1997 (named as basic value transfer), are advantageous in ESV research at large spatial scales (such as at national and regional scales) [8]. They used unit values to evaluate global ecosystems using per-unit-area monetary values of ecosystem services. Different from Costanza’s method, Xie and his colleagues used an expert-based scoring system to assign scores to ecosystem services by ecological experts and developed a matrix of equivalent factors for ESV assessment in China (known as expert modified value transfer) [9]. Costanza’s method and its variants have assessed ecosystem services and developed unit values for ecosystem services based on published case studies. The UV methods improved by Xie and his colleagues are based on scores of ecological experts through questionnaire surveys. Costanza’s and Xie’s methods have been widely applied across the world, thanks to their ease in calculating and aggregating ESVs at large spatial scales [29–31].

Recently UV methods have received global attention in ESV assessment, there however still exist significant issues. Land use and land cover classification, a critical factor used in UV methods, is limited to a few categories or types. As a result, ecological reality is oversimplified [32]. Furthermore, equivalent factors of water areas in current UV methods are set much higher than those of other land use and land cover types [33,34]. For example, Xie’s studies assumed equivalent factors of “hydrological regulation” and “water resources” services within water areas to be approximately 120 and 10 times the unit value of food production from dry areas of arable land, respectively. It is worth noting that equivalent factors of ecosystem services significantly governed accuracy of UV methods [34,35]. Additionally, existing UV methods only provide a static ESV picture of ecosystem services and adopt a unified system of equivalent factors for spatial aggregation. They ignore temporal changes of ecosystem services and neglect spatiotemporal heterogeneity of study areas [16].

In addressing the above-mentioned issues, researchers have improved UV methods by revising or refining equivalent factors for various ecological communities and regions [10,36–38]. Some scholars have considered built-up land when evaluating urban ESVs. In China, Deng first proposed equivalent factor of built-up land [39]. This equivalent factor was subsequently applied in the evaluation of ESVs in cities [40,41]. Additionally, adjustment factors were introduced in UV methods to consider resource supplies. These adjustment approaches included net primary productivity (NPP) [33], normalized difference vegetation index (NDVI) [42], precipitation and fractional vegetation [43]), socioeconomic development (including ability to pay [44], willingness to pay[25], and marginal value corrections [45]), and resource scarcity (including population density [46] and per capita natural resources [37]).These approaches have considered biotic communities with equivalent factors and improved adjustment models for equivalent factors. However, there are two main weaknesses in these studies. First, the equivalent factor methods are more suitable for natural ecosystems with insufficient consideration of artificial ecosystems. Second, the current spatiotemporal adjustment factors only consider external spatial heterogeneity and ignore internal spatial heterogeneity. Therefore, it is necessary to supplement equivalent factors of artificial ecosystems, improve multidimensional and multi-level spatiotemporal adjustment factors, and construct a new method for evaluating urban ESVs.

To address these issues, this paper describes a new method to evaluate urban ESVs from the perspective of artificial ecosystem. By considering both artificial and natural ecosystems, our study transcends the traditional focus on natural ecological communities and explores impacts of artificial ecosystems on urban development.

## Study area

**Fig 1:**
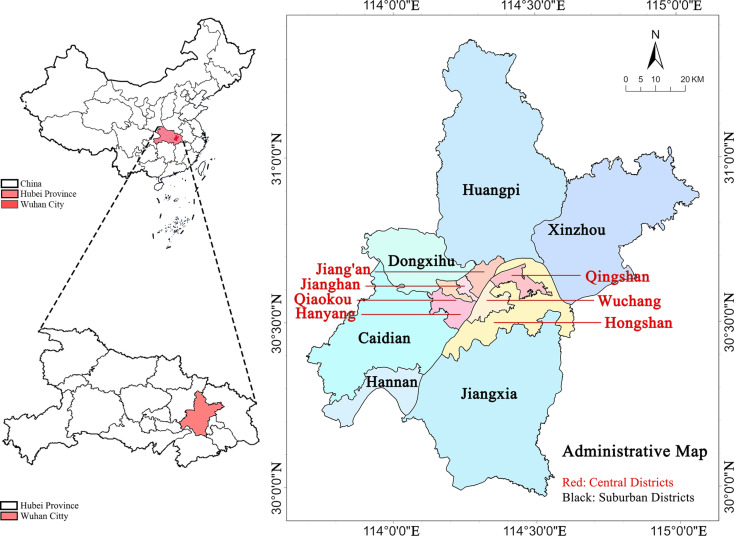
Geographical location of Wuhan, China(This figure is similar but not identical to the topographic map in 2018, and is therefore for illustrative purposes only).

Wuhan is the capital of Hubei Province, a very important city in China and a place to meet the Yangtze River and the Han River (Fig 1). The total area and the population of Wuhan is 8569 km^2^ and 11.08 million, respectively, with an urbanization rate of 80.29% as of 2018. The Gross Domestic Product (GDP) puts Wuhan the third richest city in China as of 2018. Wuhan has 13 districts, among which seven are downtown districts and six are new urban districts. The city is a transportation hub of China and has been rated as one of the three cities with the richest inland wetland resources in the world by the National Geographic magazine.

The elevation in the city ranges from 19.2 m to 873.7 m. Most portions of the city are below 50 m. The primary geomorphic type is plain, which accounts for 39.3% of the land in Wuhan. Wuhan has a typical subtropical monsoon climate. The annual average temperature ranges 15.8–17.5 °C and the frost-free season lasts for 211–272 days. The annual precipitation is 1150–1450 mm, while the rainfall season is concentrated in July and August. The annual duration of sunshine is 1810–2100 hours, and the total annual radiation is 104–113 kcal/cm^2^ [Wuhan almanac 2019].

The monthly average precipitation in Wuhan is between 21.4–238.6mm, with the highest precipitation in June and the lowest one in December. The average precipitation accounts for 18% and 2% of the annual precipitation, respectively. The annual average temperature in Wuhan ranges from 16.3 °C to 16.8 °C. The lowest and highest temperatures are -8.8 °C and 39.3 °C, respectively. The annual sunshine hours in Wuhan range from 1664.3 hours to 2002.2 hours. The average monthly wind speed in Wuhan is mostly around 2m/s, and the maximum wind speed in each month exceeds 10m/s [Wuhan City Chronicles].

## Methodology

### Research framework

Compared with natural ecosystems, urban ecosystems exhibit characteristics such as predominantly artificial environments, dense populations, and spatial heterogeneity [28,35,46]. The previous researches cannot easily address urban ecosystems such as built-up area dominating cities and have limitations. This study, recognizing this issue, revised existing model in two ways and made it applicable for cities. Firstly, a set of equivalent factors are introduced in our UV model combined with artificial ecosystem determined by meta analyses and other factors for natural ecosystem by Xie’model. Also, a set of adjustment factors are developed to further fine tune our UV model for reflecting temporal dynamics and spatial heterogeneity. Secondly, four key driving forces characterized by demographic drivers, economic drivers, industrial drivers and natural drivers were chosen, which highlighted the factors of industry and population, strengthening the human dominated characteristics of urban ecosystems.

**Fig 2:**
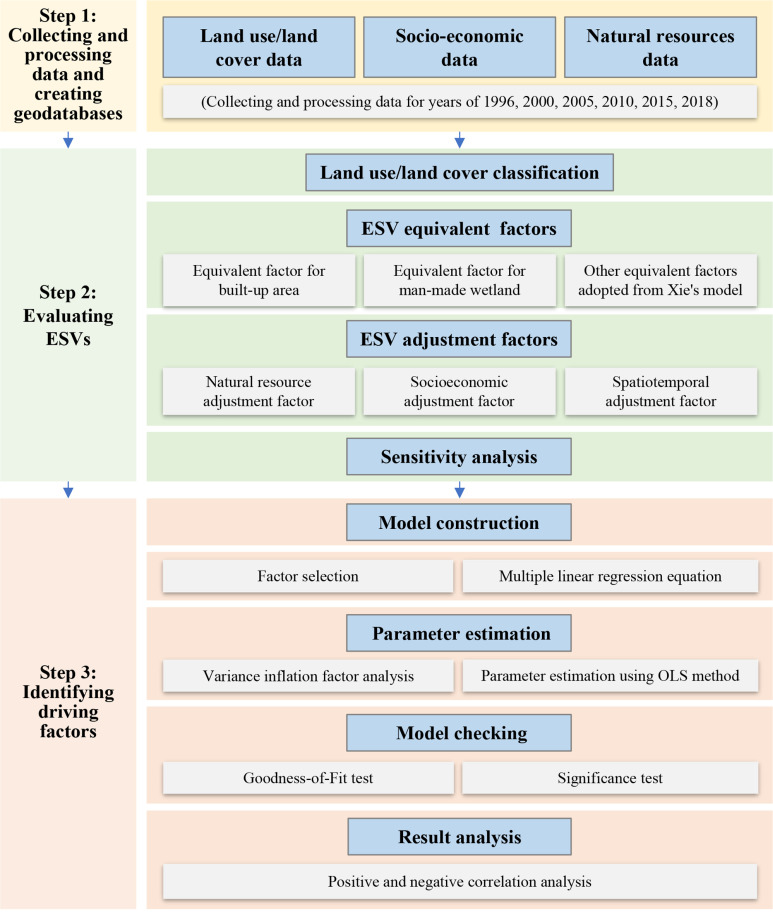
The integrated evaluation methodology and technical flow chart used in this study.

In this study, UV model and OLS model have been utilized to evaluate spatiotemporal changes of ESV and identify driving factors affecting ESV changes, respectively. The research framework specifically includes three steps (Fig 2): 1) collecting and processing data as well as creating geodatabases. Datasets driven by land use data socioeconomic data and natural resources data were included. This study utilized historical land use data to conduct a long-term analysis of temporal changes in Wuhan from 1996 to 2018. 2) developing UV model. Firstly, built-up land and artificial wetlands were increased to expand the commonly used seven types to nine types. Secondly, the equivalent factors for urban ecosystem services were determined based on the ecological substrate, combined with equivalent factor for built-up area and man-made wetland we obtained through meta-analyses, and other equivalent factors adopted from Xie’s model. Finally, the adjustment factors were selected based on the spatial feature, which include natural resources, socio-economic factors, and spatiotemporal factors. Sensitivity analysis of ESVs was also conducted to assess the effect of an equivalent factor on an ESV for a given land use and land cover type. 3) identifying driving factors that contribute to ESV changes. After selecting appropriate influencing factors, an OLS model was constructed to analyze the main influencing factors, including positive and negative coefficient analysis.

### Evaluation metrics and data collection

#### Evaluation metrics.

(1) Influencing factors of ESV evaluation

The indicators for ESV evaluation mainly include natural resource factors, socio-economic factors, as well as land use and land cover types. Natural resource and socio-economic factors reflect the spatiotemporal heterogeneity of ESs [26]. Land use and land cover types are one of the main indicators influencing ESV, which directly affects ESV changes as proxies of ESs [8,47]. Unlike natural ecosystems, urban ecosystems also include artificial ecosystems. Thus we optimized land use and land cover classification of Wuhan city.

Table 2 lists nine land use and land cover types for ESV assessment. It is noted that build-up area and man-made wetland prevail in Wuhan’s urban ecosystems. Unfortunately, previous UV models did not address these two types in ESV assessment. In this study they are included in our ESV assessment. Recognizing the nature of urban ecosystem services, we have subdivided arable land into two types: dry land and paddy field. Also, we have classified water bodies into three categories: rivers/lakes, natural wetlands (including marshes, mudflats, and reed lands), and man-made wetlands (such as reservoirs, ponds, ditches). This classification is consistent with the Ramsar Convention and the Chinese National Technical Regulations for Wetland Resources Survey and Monitoring.

**Table 2:** Definitions of land use and land cover types.

Land use and land cover types	Definition
**Arable land: dry land**	Land where crops are grown. It includes dry land, irrigated land, and garden area
**Arable land: paddy field**	Paddy field
**Woodland**	Land where trees and shrubs grow
**Grassland**	Natural grassland, artificial grass land, and meadow
**Water: river/ lake**	River and lake
**Water: natural wetland**	Water body including mudflat, marsh, and reed land
**Water: man-made wetland**	Man-made water body including reservoir, pond, aquaculture water area, and ditch, etc.
**Built-up area**	Land used for industrial, residential, commercial, and transportation purposes, etc.
**Unused land**	Unused land including sandy land, bare land, and area of saline-alkali soil

(2) Drivers of ESV changes

The changes of ESV are the results of multiple related factors, mainly including social drivers, economic drivers and natural drivers [28,38]. Due to rapid urbanization, human activities significantly impact ecosystem services. Therefore, demographic drivers are used as the representative indicators of social factors, including population and urbanization rate. The development of agricultural industry has an obvious influence on the provision services of urban ecosystems. Therefore, this paper separated industrial drivers from economic factors and comprehensively analyzed the affecting factors.

The evaluation metrics of this paper are explained in Table 3.

**Table 3:** Evaluation metrics for ESV and drivers.

ESVs		Driving forces	
ESV	ecosystem service value (¥/yr)	β	the intercept of the model
Aki	the area of LULC type k for ES i(ha)	Xi	driving factor i,
D	the unit value of the baseline ecosystem service(¥/ha)	*α*i	the coefficient of driving factor i
Efki	the equivalent factor of ecosystem service i for LULC type k	X1	Total registered residence population (10,000 persons)
NR	the natural resources adjustment factor	X2	Non-agricultural population (10,000 people)
SE	the socioeconomic adjustment factor	X3	Urbanization rate (%)
S	the spatiotemporal adjustment factor	X4	Population density (person/km2)
Ni	the average net primary productivity (NPP) (t/km^2^)	X5	GDP (10,000 yuan)
N	the average national NPP (t/ km2)	X6	Added value of primary industries (10,000 yuan)
LGDPt	Per Capita Disposable Income of Wuhan in year t(¥ per person)	X7	Added value of secondary industries (10,000 yuan)
NGDPt	Per Capita Disposable Income of China in year t(¥ per person)	X8	Added value of tertiary industries (10,000 yuan)
DYPHdisti,t	the food production per unit area of district i in year t(kg/ha)	X9	GDP per capita (10,000 yuan/person)
SEF	the sensitivity of an equivalent factor	X10	Investment in fixed assets for the whole society (10,000 yuan)
EForig,k	the original equivalent factor for LULC type k	X11	Per capita disposable income of rural residents (yuan/person)
EF-50%,k	the 50% reduction of EForig,k,	X12	Per capita disposable income of urban residents (yuan/person)
EF + 50%,k	the 50% increase of EForig,k	X13	Total agricultural output value (100 million yuan)
Ym	the ESVs of ecosystem service m(¥/yr)	X14	Total output value for forestry (100 million yuan)
		X15	Total output value of fisheries (100 million yuan)
		X16	Grain crop output (tons)
		X17	Vegetable output (tons)
		X18	Average annual rainfall (mm)
		X19	Water resources (100 million m3)

#### Data collection.

Two types of dataset were adopted in this study, which are listed as follows:

(1) Spatial datasets. They include land use and land cover data collected from two Chinese national land surveys and the Wuhan Natural Resources and Planning Bureau (WNRPB) for the years of 1996, 2000, 2005, 2010, 2015, and 2018, respectively. The spatial resolution of the land use and land cover data is less than 2.5 meters, which ensures the accuracy of the land use and land cover area (that is, Aki in Equation 1) and the ESV estimation. Data associated with national, provincial, and municipal boundaries are provided by the Geospatial Data Cloud (GDC) site (http://www.gscloud.cn).
(2) Nonspatial datasets. The socioeconomic data contain information about population, income, gross domestic product (GDP), urbanization rate, and agricultural yields. They were acquired from the Wuhan Statistics Bureau for years of 1996, 2000, 2005, 2010, 2015, and 2018, respectively (Wuhan Statistics 2019). The natural resource data include information such as annual rainfall, water resources, lakes, rivers, and wetlands. They were provided by the Wuhan Water Affairs Bureau and the Wuhan Lake Bureau. The natural resource data for the years of 1996, 2000, 2005, 2010, 2015, and 2018 were synchronized with the socioeconomic data.

A set of geodatabases thus were built to synchronize socioeconomic characteristics, natural resources, and land use and land cover data together. Also, these geodatabases were linked to the districts of Wuhan.

### Ecosystem service value assessment

#### UVmodeling.

Eleven ecosystem services (agricultural production (AP), raw material (RM), water resources (WS), gas regulation (GR), climate regulation (CR), environmental purification (EP), hydrology regulation (HR), soil retention (SR), nutrient cycling (NC), biodiversity conservation (BC), aesthetic landscape (AL)) from Wuhan’s urban ecosystems are used in our UV model, as follows:


$$\begin{array}{c} ESVs=\sum_{i=1}^{11}A_{ki}\times D\times EF_{ki}\times NR\times SE\times S \end{array}$$ (1)

Where ESVs is the ecosystem service values of eleven ecosystem services (¥/yr); A_ki_ is the area of land use and land cover type *k* for ecosystem service *i*(ha),D is the unit value of the baseline ecosystem service or the monetary value of food production per unit area of arable land (¥/ha). This unit value is adopted from the studies of Costanza et al. (1997) and Xie et al. (2008) which considered the cropland ecosystem as the representative ecosystem in most Asian countries and used the food production service of farmland as the baseline ecosystem service; EF_ki_ is the equivalent factor of ecosystem service *i* for land use and land cover type *k*; NR and SE are the natural resources and socioeconomic adjustment factors, respectively; S is the spatiotemporal adjustment factor.

The monetary value per unit area of the baseline ecosystem service (D), equivalent factors (EF), and adjustment factors for natural resources, socioeconomic, and spatiotemporal heterogeneity (NR, SE,S) in Equation 1 are described below:

#### Monetary value per unit area of the baseline ecosystem service (D).

According to the study of Xie et al. (2015), the monetary value of the baseline ecosystem service (or the food production of arable land) in China was 3,406.50 yuan/hm^2^. In addition, the average crop production per hectare per year was 5577.87 kg/hm^2^ in Wuhan and 4,891.84 kg/hm^2^ in China from 1996 to 2018. The ratio of Wuhan to China in terms of the food production per hectare was assumed in this study to be 1.1402. Therefore, D in Equation 1 is 3884.09 yuan/hm^2^.

It is important to note that arable land is not a good baseline ecosystem service in a metropolitan city like Wuhan where its land has been already transformed for various urban activities, not for activities of food production. The monetary value of food production from 1 hectare of cropland therefore cannot be used as a good base for urban ecosystem services. Therefore, it should be amended for urban ecosystem services.

#### Equivalence factors (EF).

(1) Equivalent factors adopted from Xie’s model

A matrix of equivalent factors was created for eleven ecosystem services on nine land use and land cover types (see Table 4). An equivalent factor is 1 for an ecosystem service if the unit value of the ecosystem service is fully equivalent to the unit value of food production from arable land. In Table 4, the equivalent factor of 0.25 for the AP ecosystem service on woodland indicates that the benefits (or the monetary values) provided by the AP service on woodland are 0.25 times those produced by the baseline ecosystem service (or the food production on arable land).

**Table 4:** Equivalent factor of ecosystem services per unit area in Wuhan.

	Provision			Regulation				Supporting			Culture
	AP	RM	WS	GR	CR	EP	HR	SR	NC	BC	AL
**Dry land**	0.85	0.4	0.02	0.67	0.36	0.1	0.27	1.03	0.12	0.13	0.06
**Paddy field**	1.36	0.09	-2.63	1.11	0.57	0.17	2.72	0.01	0.19	0.21	0.09
**Woodland**	0.25	0.58	0.30	1.90	5.67	1.67	3.84	2.31	0.18	2.10	0.92
**Grassland**	0.30	0.45	0.25	1.56	4.12	1.36	3.02	1.90	0.15	1.73	0.76
**River/ lake**	0.80	0.23	8.29	0.77	2.29	5.55	102.24	0.93	0.07	2.55	1.89
**Natural wetland**	0.51	0.50	2.59	1.90	3.60	3.60	24.23	2.31	0.18	7.87	4.73
**Man-made wetland**	0.50	0.36	3.42	1.75	3.49	3.02	15.75	0.90	0.15	6.77	4.26
**Built-up area**	-0.01	0.03	-0.34	-0.56	0.01	-2.50	-5.63	0.02	-0.20	0.10	0.11
**Unused land**	0.00	0.00	0.00	0.02	0.00	0.10	0.03	0.02	0.00	0.02	0.01

Note: AP-agricultural production; RM-raw material; WS-water resources; GR-gas regulation; CR-climate regulation; EP-environmental purification; HR-hydrology regulation; SR-soil retention; NC-nutrient cycling; BC-biodiversity conservation; AL-aesthetic landscape.

The equivalent factors for dry land, paddy field, river/lake, natural wetland, and unused land were adopted directly from Xie’s model [10]. The equivalent factor for woodland was the average of Xie’s equivalent factors for coniferous and broad-leaved forests. In addition, the equivalent factor for grassland was the average of Xie’s equivalent factors for shrubs and grasslands. It is important to point out that the equivalent factors for the ecosystem services on build-up area and man-made wetland were not available in previous UV models [40,44,47,48]. These equivalence factors were created in this study and are described below.

(2) Equivalent factor of man-made wetland

The unit values of ecosystem services on man-made wetlands were identified and analyzed from previous studies [22,49–53]. Table 5 provides a list of the unit values of man-made wetlands in relation to natural wetlands. For example, the ratio of the AP service is 1.05 in Chen and Zheng’s study. This ratio means that the benefits (or the monetary values) provided by the AP service on man-made wetland are 1.05 times those produced by the same AP service on natural wetland. This ratio was not used directly for our study because Chen and Zheng’s study did not provide the ratio for WC and NC services.

**Table 5:** Ratio of equivalent factor of man-made wetland to natural wetland.

Research object	Provision			Regulation				Supporting			Culture	Reference
	AP	RM	WS	GR	CR	EP	HR	SR	NC	BC	AL	
**Wuhan**	1.05	1.05		1.01	1.00	1.01	1.03	0.05		0.91	0.24	[49]
**Bengbu**	2.19			1.37		0.00	0.05			1.00	0.00	[50]
**Hanshiqiao**	0.85	0.85	0.85	0.85	0.85	0.85	0.85	0.85	0.85	0.85	1.95	[51]
**Shanghai**	0.51				0.18	0.06	0.04	0.28		0.18	0.84	[52]
**Beijing**			1.79		1.78	1.84	1.20			2.16		[22]
**Haikou**	0.34	0.23		0.46	0.08	0.44	0.73	0.00		0.03	0.58	[53]
**Average**	0.99	0.71	1.32	0.92	0.97	0.84	0.65	0.39	0.85	0.86	0.90	

Using the average of the ratios for each ecosystem service in Table 5, we determined the equivalent factors of ecosystem services on man-made wetland in relation to those of the baseline ecosystem service. As a result, the equivalent factors for the man-made wetland were 0.50 (AP), 0.36 (RM), 3.42 (WS), 1.75 (GR), 3.49 (CR), 3.02 (EP), 15.75 (HR), 0.90 (SR), 0.15 (NC), 6.77 (BC), and 4.26 (AL), respectively (see Table 4). Take the AP service as an example. The equivalent factor of 0.50 was calculated by 0.99 (the ratio of man-made wetland to natural wetland in Table 4) * 0.51 (the equivalent factor of the AP service on natural wetland in Table 4). This equivalent factor (0.5) means that the benefits (or the monetary values) provided by the AP service on man-made wetland are 0.5 times those produced by the baseline ecosystem service. These equivalent factors for the man-made wetland provide a theoretical base and a practical reference in assessing urban ESVs in regions experiencing rapid urbanization.

(3) Equivalent factor of built-up area

To date, Xie et al. (2008, 2015) and many other studies have assigned an equivalence factor of 0 to the ecosystem services on built-up areas. In this study the unit values of ecosystem services on built-up areas were identified and analyzed from previous studies [39,54–64] (see Table 6). All the unit values were then averaged to be the equivalent factor of built-up areas. For the ecosystem services of AP, RM, WS, GR, CR, EP, HR, SR, NC, BC, and AL, the equivalent factors of built-up areas were calculated to be -0.01, 0.03, -0.34, -0.56, 0.01, -2.50–5.63, 0.02, -0.20, 0.10, and 0.11 respectively. It is noted that the equivalent factor of the AP service on built-up areas is negative (-0.01), which means that the benefits (or the monetary values) provided by the AP service on built-up areas are less than those produced by the baseline ecosystem service.

**Table 6:** Equivalence factor of built-up lands in China (yuan/hm^2^).

Research object	Provision			Regulation				Supporting			Culture	Reference
	AP	RM	WS	GR	CR	EP	HR	SR	NC	BC	AL	
**Chengdu, Sichuan**	0	0	0	0	0	-2.28	-5.2	0	0	0	0	[54]
**Dehua, Fujian**	0	0	0	-2.42	0	-2.46	-7.52	0	0	0	0.09	[55]
**Xianyang, Shanxi**	0	0	0	0	0	-2.34	-9.7	0	0	0	0	[56]
**Henan**	0.28	0.3	-3.71	0	0	-2.46	-5.35	0.02	0	0.34	1.2	[57]
**Qinba, Sichuan**	0	0	0	0	0	-1.53	-4.69	0	0	0	0	[58]
**Shunde, Guangdong**	0.01	0.03	-0.34	0.11	0.1	0.31	0.21	0.13	0.01	0.12	0.05	[59]
**Hubei**	0	0	0	0	0	-2.46	-7.55	0	0	0	0	[60]
**Qinglong, Hebei**	0.01	0	0	0	0	-2.46	-7.51	0.02	0	0.34	0.01	[61]
**Gaoyang, Hebei**	0	0	0	-1.6	0	-3.71	-3.48	0	0	0	0	[62]
**Changsha, Hunan**	0	0	0	-0.22		-9.09	-4.24	0	0	0	0	[63]
**Wuhan, Hubei**	-0.38	0	0	-0.22	0	-1.56	-5.02	0	0	0	0	[64]
**Tiantai, Zhejiang**	0.01	0	0	-2.42	0	0	-7.51	0.02	-2.46	0.34	0.01	[39]

#### Adjustment factors (NR, SE,S).

(1) Adjustment factor for natural resources (NR)

Natural resources-based ecosystem services (including gas regulation, climate regulation, soil retention, environmental purification, etc.) have more impact on their monetary values to human well-being than the baseline ecosystem service. These ecosystem services need to be further adjusted from the monetary value per unit area of baseline ecosystem service (D).

In this study, biological productivity, which represents outcomes of regulating and supporting services [6,47] (Costanza et al. 2014; Xie et al. 2017), was used to determine the adjustment factor for natural resources. Net Primary Productivity (NPP) is a very important ecological indicator that reflects the productivity and energy conversion efficiency of an ecosystem. NPP refers to the net amount of photosynthetic organic matter absorbed by vegetation per unit area in a unit time, that is, the total amount of organic matter fixed by plant net photosynthesis minus the total amount of organic matter consumed by plant respiration. This indicator has been used as an adjustment factor in previous studies [33]. In this study, NPP is adopted to quantify the benefits of natural resources. The adjustment factor (NR) is defined as follows:


$$\begin{array}{c} \;NR=\frac{N_{i}}{N} \end{array}$$ (2)

where Ni is the average net primary productivity (NPP) (t/km^2^) in city i; N is the average national NPP (t/km^2^).

By averaging the adjustment factors of previous studies [65,66], we chose 1.01 as the natural resources’ adjustment factor for the city of Wuhan. It is noted that the adjustment factor is constant in this study. If national and city-level NPP data are available year by year, annual NR adjustment factors can be determined using Equation 2.

(2) Adjustment factor for socioeconomic activities (SE)

The monetary value per unit area of the baseline ecosystem service (D) was further adjusted for Wuhan based on the ability of people to pay for various socioeconomic (SE) activities, as follows:


$$\begin{array}{c} {SE}_{t}=\;\frac{{LGDP}_{t}}{{NGDP}_{t}} \end{array}$$ (3)

where LGDP_t_ is Per Capita Disposable Income of Wuhan in year *t* (¥ per person) NGDP_t_ is Per Capita Disposable Income of China in year *t* (¥ per person).

The SE adjustment factors for the years of 1996, 2000, 2005, 2010, 2015, 2018 were calculated to be 1.08, 1.08, 1.05, 1.11, 1.17, and 1.21, respectively.

(3) Adjustment factors for spatiotemporal heterogeneity (S)

The spatiotemporal heterogeneity inside the city of Wuhan was addressed by a set of spatiotemporal adjustment factors, which were calculated as follows:


$$\begin{array}{c} S_{{dist}_{i},\;t}\;=\frac{{DYPH}_{{dist}_{i},\;t}}{\sum_{1}^{13}\sum_{1}^{6}{DYPH}_{{dist}_{i},\;t}} \end{array}$$ (4)

Where DYPHdisti,t is the food production per unit area of district i in Wuhan in year t (kg/ha), $S_{{dist}_{i},\;t}$ is the spatiotemporal adjustment factor of district i in Year t.

The spatiotemporal adjustment factors were calculated for the districts in Wuhan. Table 7 shows the adjustment factors of all the districts in the years of 1996, 2000, 2005, 2010, 2015, and 2018. It is noted that the D in Equation 1 was transferred from its national value to the value for Wuhan based on the food production per unit area.

**Table 7:** Spatiotemporal adjustment factors.

District		1996	2000	2005	2010	2015	2018
**Suburban districts**	Dongxihu	1.04	0.97	1.01	1.07	1.08	1.03
	Hannan	1.04	0.94	1.18	1.1	0.97	1.02
	Caidian	0.96	0.97	1.08	1.06	0.98	0.9
	Jiangxia	0.95	0.9	0.98	0.95	1.11	1.02
	Huangpi	1.01	1.03	1.01	1.01	1.12	1.11
	Xinzhou	1.04	1.09	0.96	0.97	0.92	0.98
**Central** **districts**	Jiangan	1.04	0.96	1.02	1.07	1.03	1.03
	Jianghan	1.04	0.96	1.02	1.07	1.03	1.03
	Qiaokou	1.04	0.96	1.02	1.07	1.03	1.03
	Hangyang	1.04	0.96	1.02	1.07	1.03	1.03
	Wuchang	1.04	0.96	1.02	1.07	1.03	1.03
	Qingshan	1.04	0.96	1.02	1.07	1.03	1.03
	Hongshan	1.04	0.96	1.02	1.07	1.03	1.03

#### Sensitivity analysis of ESVs.

Sensitivity of equivalent factor (SEF), widely adopted in previous ESV studies [35,67], was used to evaluate the effect of an equivalent factor on an ESV for a given land use and land cover type. SEF is measured below by ESV changes (in percentage) over the change of the equivalent factor (in percentage):


$$\begin{array}{c} SEF=\left|\frac{\frac{{ESVs}_{-50\%,\;\;\;k}-{ESVs}_{orig,k}}{{ESVs}_{orig,k}}}{\frac{{EF}_{-50\%,\;\;\;k}-{EF}_{orig,k}}{{EF}_{orig,k}}}\right| \end{array}$$ (5-a)

or


$$\begin{array}{c} SEF=\left|\frac{\frac{{ESVs}_{+50\%,\;\;\;k}-{ESVs}_{orig,k}}{{ESVs}_{orig,k}}}{\frac{{EF}_{+50\%,\;\;\;k}-{EF}_{orig,k}}{{EF}_{orig,k}}}\right| \end{array}$$ (5-b)

Where SEF is the sensitivity of an equivalent factor. EF_orig,k,_ EF_-50%,k_, and EF_+50%,k_ are the original equivalent factor for land use and land cover type *k*, the 50% reduction of EF_orig,k,_ and the 50% increase of EF_orig,k_. For an example, EF_orig, dry land_ is 0.85 for the AP ecosystem service. EF_-50%, dry land_ is 0.425 for the AP ecosystem service, while EF_+50%, dry land_ is 1.275 for the AP ecosystem service.

By setting up ±50% of the equivalent factor for each land use and land cover type, we calculated the changes in ESVs. It is noted that the SEF is inelastic if 0 < SEF ≤ 1 and is elastic if SEF > 1. The inelastic SEF indicates that ESVs is not sensitive to the equivalent factor. It also indicates that the closer the value of SEF is to zero, the less sensitive the ESVs is in response to the equivalent factor. The elastic SEF however demonstrates that ESVs are closely related to its equivalent factor.

### Driving force analysis

Once ESVs were calculated, our next step was to identify driving factors which govern the changes of ESVs in the city of Wuhan. ESV was chosen as the dependent variable, and a set of 19 possible driving factors were selected as independent variables for investigation (see Table 3). Pearson’s correlations between the ESVs and the driving factors were calculated using a multiple regression model in Stata 16.0. The multiple linear regression model was defined below:


$$\begin{array}{c} Y_{m}=\beta +\alpha_{i}\sum_{i=1}^{n}X_{i} \end{array}$$ (6)

Where Y_m_ denotes the ESVs of ecosystem service *m*, *β* is the intercept of the model, X_i_ denotes driving factor *i*, and α_*i*_ denotes the coefficient of driving factor *i*. All the driving factors are listed in Table 3.

In coefficient estimation, the inflation coefficient of variance (VIF) was used to measure the degree of multicollinearity, which can reflect the degree to which the independent variable is influenced by other independent variables. The larger the VIF value, the more severe the multicollinearity. The driving factors with severe multicollinearity were excluded according to the principle of VIF > 0.75. Then, we further adopted Ordinary Least Squares (OLS) to estimate the regression coefficients α and β in Stata 16.0. The regression coefficient α greater than 0 indicates that the factor has a positive impact on ESV, while α less than 0 indicates a negative impact on ESV.

After estimating the coefficients, various significance tests are performed on the model, including Goodness of Fit tests, significance tests, etc. A commonly used coefficient R² in fitting degree testing measures the goodness of fit of regression models to data. It represents the proportion of variation in the dependent variable that can be explained by the independent variable, with a range of values from 0 to 1. The closer it is to 1, the better the fitting effect. P-value is the significance test result of a parameter, which is generally divided into three levels of significance testing: P ≤ 0.01, 0.01 < P ≤ 0.05, 0.05 < P ≤ 0.1. The smaller the P-value, the more significant it is.

## Results

### Land use and land cover changes

The land use and land cover changes in Wuhan during 1996–2018 were investigated using the GIS technology. Table 8 shows that Wuhan experienced a significant land transformation process in built-up area. As a result of this transformation or urbanization, the build-up area in 2018 was expanded to be approximately 1.64 times that in 1996, accounting for the largest proportion (24.41%) of the land in Wuhan. The built-up areas were stretched out gradually from the core of the city to the suburban areas. The land use and land cover dynamic index of build-up area was 2.89% (see Table 9), showing the most increasing annual change of land use and land cover in Wuhan.

**Table 8:** Land use and land cover changes in Wuhan (1996-2018).

Land use and land cover categories	Area (hm^2^)						Change rate (%)					
	1996	2000	2005	2010	2015	2018	1996-2000	2000-2005	2005-2010	2010- 2015	2015- 2018	1996- 2018
**Paddy field**	232424	229036	203638	149370	141773	139784	-1.46	-11.09	-26.65	-5.09	-1.40	-39.86
**Dry land**	191904	188737	157486	175703	164556	161204	-1.65	-16.56	11.57	-6.34	-2.04	-16.00
**Woodland**	77584	77784	101566	105739	103585	102573	0.26	30.58	4.11	-2.04	-0.98	32.21
**Grassland**	6901	6901	283	2	2	2	0.00	-95.90	-99.24	0.00	0.00	-99.97
**River/lake**	104530	104621	112853	110734	110255	110117	0.09	7.87	-1.88	-0.43	-0.13	5.34
**Natural wetland**	24400	24304	24504	16956	16819	16740	-0.39	0.82	-30.80	-0.81	-0.47	-31.39
**Man-made wetland**	70202	71810	90230	122591	115822	113538	2.29	25.65	35.87	-5.52	-1.97	61.73
**Built-up area**	127926	132954	153111	171266	200218	209182	3.93	15.16	11.86	16.91	4.48	63.52
**Unused land**	21044	20768	13244	4554	3885	3775	-1.31	-36.23	-65.62	-14.68	-2.85	-82.06

**Table 9:** Land use and land cover dynamic index (K) in Wuhan (1996-2018).

Land use and land cover categories	Percentage of land use type (%)						Land use and land cover dynamic index K (%)					
	1996	2000	2005	2010	2015	2018	1996-2000	2000-2005	2005-2010	2010- 2015	2015- 2018	1996- 2018
**Paddy field**	27.12	26.73	23.76	17.43	16.54	16.31	-0.36	-2.22	-5.33	-1.02	-0.47	-1.81
**Dry land**	22.39	22.03	18.38	20.50	19.20	18.81	-0.41	-3.31	2.31	-1.27	-0.68	-0.73
**Woodland**	9.05	9.08	11.85	12.34	12.09	11.97	0.06	6.11	0.82	-0.41	-0.33	1.46
**Grassland**	0.81	0.81	0.03	0.00	0.00	0.00	0.00	-19.18	-19.86	0.00	0.00	-4.54
**River/Lake**	12.20	12.21	13.17	12.92	12.87	12.85	0.02	1.57	-0.38	-0.09	-0.04	0.24
**Natural wetland**	2.85	2.84	2.86	1.98	1.96	1.95	-0.10	0.16	-6.16	-0.16	-0.16	-1.43
**Man-made wetland**	8.19	8.38	10.53	14.31	13.52	13.25	0.57	5.13	7.17	-1.10	-0.66	2.81
**Built-up area**	14.93	15.52	17.87	19.99	23.36	24.41	0.98	3.03	2.37	3.38	1.49	2.89
^**Unused land**^	2.46	2.42	1.55	0.53	0.45	0.44	-0.33	-7.25	-13.12	-2.94	-0.94	-3.73

Note: $K=\frac{A_{target}-A_{initial}}{A_{initial}}\times \frac{1}{T}\times 100\;\backslash \%$, *K* represents a dynamic index for a single land use and land cover type. A_target_ and A_initial_ are the area (hm^2^) of the land use and land cover type at the target and initial year of the study period, respectively, and *T* is the study period (year). If *T* is one year, *K* refers to the annual change rate.

In 1996, paddy field, which provides the baseline ecosystem service, accounted for the largest proportion of land (27.12%). However, it decreased sharply (-39.86% in Table 8) in 2018 and became the third largest area. Its land use and land cover dynamic index was -1.81% (see Table 9). Additionally, man-made wetland and woodland increased sharply first, reached their peak in 2010, and then decreased slightly.

It is worth noting that a significant increase in forest land (30.58%) and a significant decrease in grassland (-95.90%) occurred between 2000 and 2005, due to Wuhan’s three-year policy of returning farmland to forests and the balanced development of non-arable land into arable land. In addition, man-made wetlands such as ponds and ditches had significantly increased by 25.65% (2000–2005) and 35.87% (2005–2010) due to the expansion of the aquaculture industry and the land consolidation projects in Wuhan. Their land use and land cover dynamic index was 2.81.

### ESVs and their change patterns

#### Ecosystem service values.

ESVs for 11 ecosystem services and nine land use and land cover types were calculated using Equation 1. Table 10 lists ecosystem service values in Wuhan from 1996 to 2018. It is observed that the ESVs for paddy field in 1996 were 3.80 billion Chinese yuan. The ESVs were the sum of the 11 ecosystem services (AP, RM, WS, GR, CR, EP, HR, SR, NC, BC, and AL). Table 11 describes ESVs by ecosystem service in Wuhan from 1996 to 2018. From Table 11, the ESVs for the AP service in 1996 were 2.67 billion Chinese yuan. This was the sum of the ESVs for the AP service from the land use and land cover types.

#### Changes in total ESVs.

**Fig 3:**
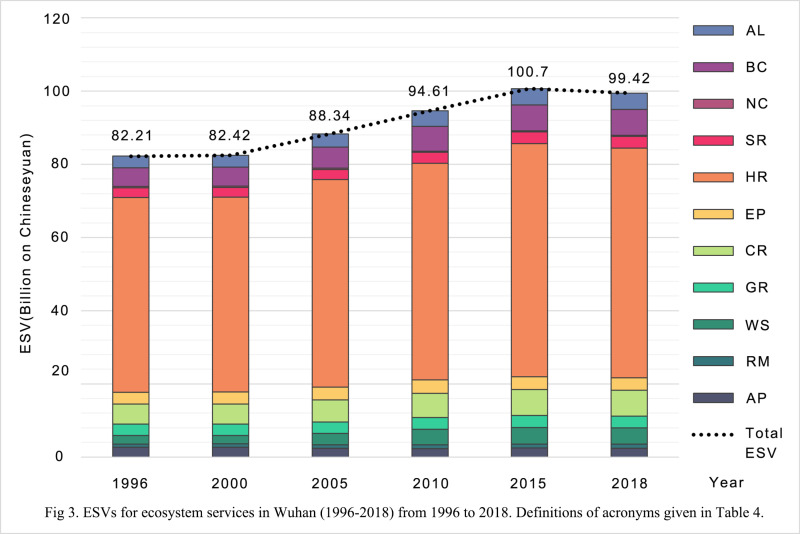
ESVs for ecosystem services in Wuhan from 1996 to 2018. **Definitions of acronyms given in Table 4**.

The total ESVs for the years of 1996, 2000, 2005, 2010, 2015, and 2018 are listed in **Table 10**, **Table 11**, and Fig 3. They increased 20.94% in response to the land use and land cover changes from 1996 to 2018. Specifically, the total ESVs increased from 82.21 billion Chinese yuan in 1996 to 100.70 billion Chinese yuan in 2015. After 2015, the total ESVs decreased to 99.42 billion Chinese yuan in 2018.

**Table 10:** ESVs by land use and land cover type in Wuhan (1996 – 2018) (billion Chinese yuan).

Land use and land cover type		Paddy field	Dry land	Wood land	Grass land	River /Lake	Natural wetland	Man-made wetland	Built-up area	Unused land	Total
**1996**	**ESVs**	3.8	3.27	7.13	0.46	54.97	5.37	12.12	-4.92	0.02	82.21
	**P(%)**	4.62	3.97	8.67	0.56	66.86	6.53	14.74	-5.98	0.02	100
**2000**	**ESVs**	3.79	3.25	7.14	0.47	54.96	5.41	12.43	-5.04	0.02	82.42
	**P(%)**	4.6	3.94	8.67	0.57	66.68	6.56	15.08	-6.12	0.02	100
**2005**	**ESVs**	3.25	2.63	8.83	0.02	58.69	5.43	15.2	-5.74	0.01	88.34
	**P(%)**	3.68	2.98	10	0.02	66.44	6.15	17.21	-6.49	0.01	100
**2010**	**ESVs**	2.52	3.1	9.5	0	60.49	3.99	21.85	-6.85	0	94.61
	**P(%)**	2.67	3.28	10.04	0	63.93	4.22	23.1	-7.24	0	100
**2015**	**ESVs**	2.69	3.14	10.35	0	66.84	4.09	22.2	-8.6	0	100.7
	**P(%)**	2.67	3.11	10.28	0	66.37	4.06	22.05	-8.54	0	100
**2018**	**ESVs**	2.66	3.11	10.3	0	66.14	4.09	22.18	-9.06	0	99.42
	**P(%)**	2.68	3.13	10.36	0	66.53	4.11	22.31	-9.11	0	100
**1996-** **2018**	**VC**	-1.13	-0.16	3.17	-0.46	11.17	-1.28	10.06	-4.15	-0.01	17.21
	**CR (%)**	-29.84	-4.87	44.49	-100	20.33	-23.88	83.06	-84.35	-77.78	20.94
	**ADD(%)**	-1.36	-0.22	2.02	-4.55	0.92	-1.09	3.78	3.83	-3.54	0.95

Note: P-Proportion (%); VC-value change (billion Chinese yuan); CR-Change Rate of ESVs (%); ADD-Annual Dynamic Degree (%).

**Table 11:** ESVs by ecosystem service in Wuhan (1996-2018) (billion Chinese yuan).

^Ecosystem^ ^Service Value^		^Provision^			^Regulation^				^Supporting^			^Culture^	^Total^
		AP	RM	WS	GR	CR	EP	HR	SR	NC	BC	AL	
**1996**	**ESVs**	2.67	0.91	2.30	3.12	5.45	3.24	53.24	2.67	0.34	5.14	3.15	82.21
	**P(%)**	3.24	1.11	2.80	3.79	6.63	3.94	64.76	3.25	0.41	6.25	3.83	100.00
**2000**	**ESVs**	2.67	0.92	2.33	3.12	5.48	3.23	53.29	2.68	0.34	5.20	3.19	82.42
	**P(%)**	3.23	1.11	2.82	3.78	6.65	3.92	64.66	3.25	0.41	6.31	3.86	100.00
**2005**	**ESVs**	2.42	0.91	3.17	3.08	6.02	3.47	56.81	2.75	0.30	5.83	3.58	88.34
	**P(%)**	2.73	1.03	3.59	3.49	6.82	3.93	64.31	3.11	0.34	6.60	4.05	100.00
**2010**	**ESVs**	2.35	1.01	4.24	3.19	6.66	3.67	59.14	3.04	0.28	6.82	4.21	94.61
	**P(%)**	2.48	1.07	4.48	3.37	7.03	3.88	62.51	3.22	0.29	7.21	4.45	100.00
**2015**	**ESVs**	2.47	1.07	4.53	3.27	7.08	3.57	63.67	3.22	0.26	7.14	4.42	100.70
	**P(%)**	2.45	1.06	4.49	3.25	7.03	3.55	63.23	3.19	0.26	7.09	4.39	100.00
**2018**	**ESVs**	2.45	1.06	4.48	3.22	7.05	3.41	62.78	3.20	0.25	7.12	4.41	99.42
	**P(%)**	2.46	1.07	4.50	3.24	7.09	3.43	63.15	3.22	0.25	7.17	4.43	100.00
**1996-** **2018**	**VC**	-0.22	0.15	2.18	0.11	1.60	0.17	9.55	0.53	-0.09	1.99	1.26	17.21
	**CR (%)**	-8.25	16.43	94.61	3.43	29.37	5.25	17.93	19.87	-26.41	38.65	40.07	20.94
	**ACR(%)**	-0.38	0.75	4.30	0.16	1.34	0.24	0.81	0.90	-1.20	1.76	1.82	0.95

Note: AP-agricultural production; RM-raw material production; WS-water resources; GR-gas regulation; CR-climate regulation; EP-environmental purification; HR-hydrology regulation; SR-soil retention; NC-nutrient cycling; BC-biodiversity conservation; AL-aesthetic landscape; ESVs-ecosystem service value (billion Chinese yuan); P -Proportion (%); VC-value change (billion Chinese yuan); CR-Change Rate of ESVs (%); ACR- Annual change rate (%).

#### Changes in ESVs by land use and land cover type.

In 1996, river/lake was the major contributor (54.97 billion Chinese yuan) to the total ESVs, accounting for 66.86%. Similarly, man-made wetland and woodland accounted for 14.74% and 8.67%, respectively. The ESVs of grassland and unused land were less than 1% (0.56% + 0.02% = 0.58%), while the ESVs provided by built-up area were negative (-4.92 billion Chinese yuan).

The largest increasing rate in ESVs is observed in man-made wetland (83.06%), followed by woodland (44.49%) and river/lake (20.33%). Grassland had its worse decreasing rate (-100.00%), followed by built-up area (-84.35%).

#### Changes in ESVs by ecosystem service.

Fig 3 shows the ESVs and their changes in 11 ecosystem services from 1996 to 2018. ESVs in regulatory services (combined) accounted for over 65% of the total ESVs each year, followed by supporting services (approximately 10%), provision services (nearly 8%), and cultural services (roughly 4%). Specifically, the ecosystem service contributing the most ESVs were hydrology regulation (over 60%), followed by biodiversity conservation (about 6%), climate regulation (about 6%), water resources (about 4%), aesthetic landscape (about 4%), environmental purification (about 3.5%), and gas regulation (about 3.2%). The proportion of the ESVs of other ecosystem services were less than 10%.

It is noted that ESVs of ecosystem services except the agricultural production (AP) and nutrient cycling (NC) services increased during the urbanization process. This observation confirmed that the ESVs in the AP and NC services declined since their areas shrank significantly during the urbanization process. On the contrary, the largest increase in ESVs is observed in hydrology regulation (9.55 billion Chinese yuan), followed by water resources (2.18 billion Chinese yuan). Additionally, water resources had the highest rate of change (94.61%) and the highest annual change rate (4.30%).

#### Spatiotemporal change patterns of ESVs.

**Fig 4:**
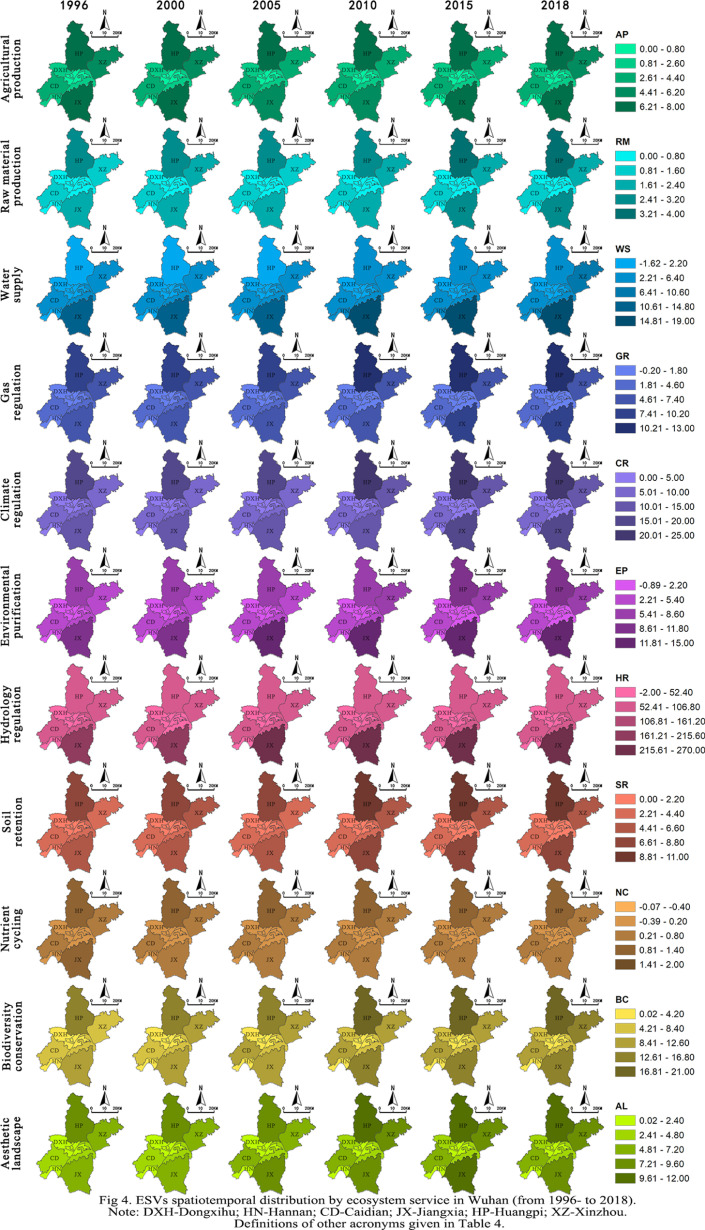
ESVs spatiotemporal distribution by ecosystem service in Wuhan from 1996 to 2018. **Note: DXH-Dongxihu; HN-Hannan; CD-Caidian; JX-Jiangxia; HP-Huangpi; XZ-Xinzhou. Definitions of other acronyms given in Table 4**.

Spatiotemporal change patterns of ESVs for 11 ecosystem services are shown in Fig 4. In the north and south of Wuhan, especially in the districts of Jiangxia, Huangpi, and Xinzhou, ESVs were higher than other districts primarily due to their expansion of man-made wetland, natural wetland, and woodland areas. ESVs in the central districts were lower than suburban districts.

It is observed that ESVs decreased from 1996 to 2018 in central districts except the Hongshan district. This finding supported the impact of increased build-up area in central districts on ESVs reduction. This finding also confirmed that the expansion of river/lake areas in the Hongshan district provided a gain of ESVs. The ESVs in suburban districts increased, especially in the Huangpi and Jiangxia districts. The ESVs provided by agricultural production and nutrient cycling continuously declined in all districts over time. However, the ESVs for hydrology regulation, climate regulation, water resources, and biodiversity conservation increased significantly in the districts of Jiangxia, Huangpi, Xinzhou, and Caidian.

#### Sensitivities of ESVs to equivalent factors (EF).

Sensitivities of ESVs to equivalent factors for each land cover type were calculated using Equation 5. As shown in **Table 12**, all the sensitivities were less than 1, which indicated that the estimated ESVs in this study were reliable even when the equivalent factors increased or decreased 50% of their original values. The sensitivity of the equivalent factor for river/lake in 2005 was 0.72, the highest among all the sensitivities in **Table 12**. This demonstrates that ESVs of Wuhan were highly sensitive to river/lake and a small change of river/lake led to a large change of ESVs. The second largest contributor to ESVs was man-made wetland. The high sensitivity values for river/lake and man-made wetland can be explained by the fact that equivalent factors from these land use and land cover types were relatively high for almost all the eleven ecosystem services. In addition, river/lake and man-made wetland accounted for a large portion of areas in Wuhan. The sensitivities related to grassland and unused land however were very low due to their low share of area in Wuhan and low equivalent factors for ecosystem services.

**Table 12:** Sensitivity of equivalent factors factors in Wuhan (1996-2018).

Land use and land cover types	1996	2000	2005	2010	2015	2018
**Paddy field**	0.0462	0.0460	0.0399	0.0245	0.0205	0.0182
**Dry land**	0.0397	0.0394	0.0324	0.0301	0.0239	0.0212
**Woodland**	0.0867	0.0866	0.1086	0.0923	0.0791	0.0702
**Grassland**	0.0056	0.0057	0.0002	0.0000	0.0000	0.0000
**River/Lake**	0.6686	0.6669	0.7214	0.5875	0.5106	0.4508
**Natural wetland**	0.0653	0.0656	0.0668	0.0387	0.0312	0.0279
**Man-made wetland**	0.1474	0.1508	0.1868	0.2123	0.1696	0.1512
**Built-up area**	0.0598	0.0612	0.0705	0.0665	0.0657	0.0618
**Unused land**	0.0002	0.0002	0.0001	0.0000	0.0000	0.0000

### ESVs driving forces

Pearson’s correlation coefficient study was conducted to address the multicollinearity problems among all the 19 potential factors. The threshold set up for eliminating a potential factor was 0.75. When the correlation coefficient was above or equal to 0.75, one of the potential factors in a pair of factors was eliminated. After Pearson’s correlation analysis, X2, X3, X5, X6, X7, X8, X10, X11, X12, X13, X14, and X17 were eliminated from further consideration in the multivariate regression model. Analysis of Variance (ANOVA) was then undertaken to further detect multicollinearity among the remining factors or variables (X1, X4, X9, X15, X16, X18, and X19). A variance inflation factor (VIF) was calculated for each remining factor. It was observed that the VIF of each remining variable was less than 3, lower than the critical value of 5. This indicates that there were no more significant multicollinearity issues among the remaining variables.

After the Pearson and ANOVA investigations, the seven remaining variables were considered in the multivariable linear regression analysis. The results of the analysis are shown in **Table 13**. The coefficients of X4, X15, X16, and X18 on Y in column (1) passed the significance test at the 1% level, with the coefficients of -0.4487, 6.6270, 0.0003, and 0.2267, respectively. This indicates that X15, X16, and X18 had a significant promoting effect on Y, but X4 had a significant inhibitory effect on Y. Similarly, coefficients for Y1 (Provision ESVs), Y2 (Regulation ESVs), Y3 (Supporting ESVs), and Y4 (Culture ESVs) are shown in Table 13. The R^2^ values in the models (columns (1) - (5)) all exceeded 0.75, indicating a good fit. The models are also represented in Table 14.

**Table 13:** Multivariant linear regression analysis of ESVs.

	(1)	(2)	(3)	(4)	(5)
	Y	Y1	Y2	Y3	Y4
**X1**	0.0498	-0.0047	0.0268	0.0233*	0.0043
	(-0.12)	(-0.16)	(-0.07)	(-1.7)	(-0.69)
	*0.04*	*0.00*	*0.04*	*0.00*	*0.00*
**X4**	-0.4487***	-0.0346***	-0.3752**	-0.0269***	-0.0120***
	(-2.73)	(-2.83)	(-2.54)	(-4.76)	(-4.66)
	*0.02*	*0.00*	*0.01*	*0.00*	*0.00*
**X8**	0	0	0	0	0
	(-1.14)	(-1.23)	(-1.12)	(-0.83)	(-1.11)
	0.00	0.00	0.00	0.00	0.00
**X9**	-0.7911	-0.0659*	-0.6354	-0.0601***	-0.0297***
	(-1.49)	(-1.68)	(-1.33)	(-3.30)	(-3.57)
	*0.05*	*0.00*	*0.05*	*0.00*	*0.00*
**X10**	0	0	0	0	0
	(-1.18)	(-1.26)	(-1.13)	(-1.37)	(-1.52)
	*0.00*	*0.00*	*0.00*	*0.00*	*0.00*
**X11**	-0.0012	-0.0001	-0.0013	0.0001	0
	(-0.72)	(-0.50)	(-0.83)	(-1.25)	(-1.39)
	*0.00*	*0.00*	*0.00*	*0.00*	*0.00*
**X12**	0.0003	0	0.0003	0	0
	(-0.73)	(-0.76)	(-0.71)	(-0.75)	(-0.63)
	*0.00*	*0.00*	*0.00*	*0.00*	*0.00*
**X14**	-0.1306	-0.012	-0.1015	-0.0128	-0.0044
	(-0.24)	(-0.30)	(-0.21)	(-0.69)	(-0.52)
	*0.05*	*0.00*	*0.05*	*0.00*	*0.00*
**X15**	6.6270***	0.5176***	5.2674***	0.6072***	0.2348***
	(-3.27)	(-3.44)	(-2.89)	(-8.73)	(-7.38)
	*0.20*	*0.02*	*0.18*	*0.01*	*0.00*
**X16**	0.0003***	0.00002***	0.0002***	0.00004***	0.00001***
	(-3.42)	(-2.7)	(-2.89)	(-13.57)	(-9.98)
	*0.00*	*0.00*	*0.00*	*0.00*	*0.00*
**X18**	0.2267***	0.0173***	0.2139***	-0.0040*	-0.0005
	(-3.34)	(-3.44)	(-3.51)	(-1.70)	(-0.46)
	*0.01*	*0.00*	*0.01*	*0.00*	*0.00*
**X19**	-2.9027	-0.0502	-2.3469	-0.4278***	-0.0779*
	(-1.12)	(-0.26)	(-1.01)	(-4.83)	(-1.92)
	*0.26*	*0.02*	*0.23*	*0.01*	*0.00*
**_cons(β)**	245.4965**	18.4569***	235.7309***	7.0176**	1.6736
	(-2.64)	(-2.67)	(-2.82)	(-2.2)	(-1.15)
	*9.30*	*0.69*	*8.36*	*0.32*	*0.15*
**N**	78	78	78	78	78
**R** ^**2**^	0.8025	0.7974	0.7581	0.9747	0.9641

Note: * represents that p value is less than 0.1, ** represent that p value is less than 0.05, and *** represent that p value is less than 0.01. Y denotes the total ESVs, Y1 denotes provision ESVs, Y2 denotes regulation ESVs, Y3 denotes supporting ESVs, Y4 denotes culture ESVs. The values in parentheses represent the T value, which is the statistical verification value of the T test, representing the probability of presenting the sample statistical results. The values in italics represent the standard deviation.

**Table 14:** Regression models.

Dependent	Regression Model
**Total ESVs**	Y = -0.4487X4+6.6270X15 + 0.00003X16+0.2267X18-245.4965
**ESVs for provision services**	Y1 = -0.0346X4-0.0659X9+0.5176X15 + 0.00002X16+0.0173X18-18.4569
**ESVs for regulation services**	Y2 = -0.3752X4+5.2674X15 + 0.0002X16+0.2139X18-235.7309
**ESVs for supporting services**	Y3 = 0.0233X1-0.0269X4-0.0601X9+0.6072X15 + 0.00004X16-0.0040X18-0.4278X19+7.0176
**ESVs for cultural services**	Y4 = -0.0120X4-0.0297X9+0.2348X15 + 0.00001X16-0.0779X19+1.6736

Note: Y: Service value, X1: Total registered residence population (10,000 persons), X4: Population density (people/km^2^), X9: GDP per capita (10,000 yuan/person), X15: Total fishery output (10^9^ yuan), X16: Grain crop output (tons), X18: Average annual rainfall, X19: Water resources (100 million m^3^).

A further analysis of the models provides us with the following findings:

1) ESVs in Wuhan are positively correlated with total fishery output, grain crop output, and total registered residence population. Fishery is the main contributor of ESVs. Thanks to this strong contribution, ESVs increased significantly as more lands were returned to lakes or reclaimed as man-made wetlands. In Wuhan, cultivated lands with low ecological value were also transformed into lakes and man-made wetlands. This transformation greatly helped increase the ESVs. Grain crop output also contributes to ESVs. As modern techniques are used to increase agricultural productivity and grain crop yields, the values of ecosystem services correspondingly climbed up.

It is interesting to note that total registered residence population only affects the ESVs for supporting services, not for other ecosystem services. This positive relationship with the supporting services confirms that human beings are the major player of supporting services such as water cycling, nutrient cycling, soil formation and retention, provisioning of habitats for animal and plant species, and production of atmospheric oxygen and CO2.

2) Population density, per capita GDP, and water resources have inhibitory effects on ESVs. In areas with high population density, construction land and built-up area prevail and often account for a large share of land. As a result, ecosystem resources per capita are decreased and the ESVs are reduced.

The growth of GDP per capita, accompanied by environmental pollution and resource consumption, affects negatively supply services, supporting services, and culture services in Wuhan. Water resources cause changes in soil conservation and ecological tourism environment and affects the ESVs of supporting services and culture services. For example, ground runoff (an important element in water resources) gets worse when more built-up lands are created during the urbanization process. The ground runoff further disrupts soil nutrient cycling, resulting in reduction of ESVs for supporting and culture services.

3) It is worth noting that average annual rainfall significantly promotes total ESVs and ESVs for supply and regulatory services, while negatively impacts on ESVs for supporting services. This finding confirms that the increasing rainfall expands the areas of rivers/lakes and creates new man-made wetlands (or ponds). Additionally, the increases in annual rainfall have helped Wuhan get more grain crops. Conversely, the increases of annual rainfall can get more hydraulic and soil erosions, resulting in the loss of soil fertility and biodiversity, the interruption of nutrient cycling, and the reduction of ESVs for supporting services.

## Discussions

In this section, detailed discussions are presented to highlight the validity of our UV method from the view of its theoretical contributions and recommendations to decision makers for land use planning, ecological protection, and ESV enrichment.

### ESV evolutionary features

This research analyzes the spatial-temporal dynamic evolution of ESV in urban area in the period of 1996–2018 based on a novel unit value method. The main land use and land cover types in Wuhan are farmland and water area, with the largest reduction in farmland area. The total ESV increased by 17.21 billion Chinese yuan in the whole research period, experienced a continuous increase from 1996 to 2015 and a slight decline after 2015. Water area and woodland accounted for over 95% of the total ESV, and contributed most to ESV. The trend of ESV changes was consistent with the findings of Liu and Yang (2021) [68] and Zhang et al. (2018) [13], but the total ESV was higher than their results since our method does consider man-made wetlands while they neglect this type. Despite the expansion of built-up area, the increase of the total ESV mainly is due to the growth of ecological land use(water area and woodland). This finding is consistent with the research on China’s wetland protection policies and return farmland to woodland [11,33,35,69]. It indicates that the implementation of ecological protection policies, especially wetland policies, is an important factor in urban ecological restoration.

### Driving forces for ESV changes

The results show that spatio-temporal changes in ESV of the study region are caused by multiple driving factors, among which socio-economic factors is the dominant driver. This finding is consistent with existing researches [22,36,69–71] We further found that industrial drivers was positively correlated with the ESV change. This indicated that the economic development in urban area improved the industrial structure and then affected the ecosystem services. Thus the region’s economic development can be in parallel with ecological protection.

The impact of population on the ESV changes had two aspects. On the one hand, total registered residence population was positively correlated with the total ESV. Population growth can promote economic growth and technological progress, thereby promoting environmental protection. On the other hand, continuous population growth has led to high population density, which has a negative impact on the ecological environment. The concentration of a large population has led to heavy environmental pressure and resource consumption, resulting in the decline of ESV.

### Policy implications

This study provides the following policy implications for land use and zonal management of man-made wetlands, woodlands and built-up areas:

1) our study indicates the importance of strengthening the protection and restoration of ecological land to improve regulating services of ecosystem. Firstly, Human intervention activities have both positive and negative influences on ESV changes in wetlands. Our strategies for wetland protection are quantity control and quality management of wetlands. On the one hand, wetland protection areas and red lines can be delineated, and wetland “occupation and replenishment balance” indicators can be controlled, thus to ensure that the number of wetlands does not decrease. On the other hand, we should strengthen wetland classification management and quality protection, promote ecological restoration and rational utilization. Secondly, Our ESV study encourages to further make unfavorable arable lands to woodlands. From the ESV point of view, the Chinese government policies of returning farmlands to forests lead to a certain degree of the restoration of woodlands. More attention should be concentrated on converting unfavorable arable lands into woodlands, while securing farmers’ land use rights in woodlands and strengthening regulations on existing woodland usage. Thirdly, our study results indicate that agricultural industry provides positive ESVs. It is therefore required that modern agriculture technologies should be utilized to yield more ESVs. Additionally, the spatial patterns of agriculture lands should be optimally utilized. In doing so, resources of cultivated land and forests can be effectively reserved and ecological functions can be sustainably utilized.
2) Diverse land use policies are needed to optimize the allocation of land resources in different regions and promote the balance between ecological benefits and socio-economic. The government can control the spread of built-up area and increase the land use efficiency of unit area to promote intensive utilization of land resources. Meanwhile, the industrial transformation and upgrade should be further promoted.
3) The ESV increased from the Central area to the peripheral region. Specific policies and strategies regarding different ecosystems should be adopted in different regions. For central districts, the government should control the population density and evacuate some of the population to the suburbs, which can alleviate the population pressure in the central area. In remote urban areas, the land use policy should focus on ecological value instead of economic growth. At the same time, feasible ecological compensation measures should be formulated to sustainable utilize ecosystem services.

### Novelty and validity of this study

The equivalent factor tables provided by Costanza et al. (1997, 2014) and Xie et al. (2008, 2015) were widely applied [6,8–10]. However, these tables are developed for natural ecosystems. They exclude human-dominated ecosystems in urban areas [16]. As 50% of the world’s population resides in urban areas, it is crucial to developing equivalent factor tables to evaluate human-dominated ecosystems [4]. This study, in response to this need, develops a new UV method for urban ESV assessment and considers the dynamic changes of artificial ecological communities including built-up areas and wetlands. This study creates a set of new equivalent factors for built-up lands and man-made wetlands. Additionally, a multi-dimensional spatiotemporal adjustment model is developed to consider spatiotemporal heterogeneity. Different from natural ecosystems like forests or grassland, urban ecosystems are composite ecosystems involving many artificial facilities, heterogeneous activities, and un-even socio-economic development. This adjustment model considers spatiotemporal variations in ESV evaluation.

## Conclusions and future work

In this study, we further improved the equivalent factor table for urban ecosystem by supplementing the factors of artificial ecosystems based on Xie’ s model. This paper analyzed the spatiotemporal evolution characteristics of ESVs in Wuhan city from 1996 to 2018 and its influencing factors by using the regression analysis method. This method is not only suitable for urban ecosystems, but can also be applicable to other complex systems.The main findings are as follows:

(1) In the period of 1996–2018, the total ESVs of Wuhan increased by 17.21 billion Chinese yuan, with the only increases occurring in river/lake, man-made wetland and woodland, which accounted for 45.78%, 41.23% and 12.99% of the increase. The hydrology regulation is primary ecosystem service in Wuhan, accounting for more than 77% of the total ESVs. Climate regulation and biodiversity conservation functions are lower, both accounting for around 7%. This result shows that the increase of ecological land (i.e., water area and woodland) can lead to ESV benefits despite the expansion of construction land in urban area. This provides important reference for the policy-making of land use structure optimization to achieving a balance between economic benefits and ecosystem services.
(2) The ESVs declined from 1996 to 2018 in central districts and increased in suburban districts especially in the districts of Huangpi and Xinzhou in the north and Jiangxia in the south, which have abundant water and forest resources. Only Hongshan district in central area has witnessed an increase in ESV due to the expansion of river and lake areas. The ESVs provided by agricultural production and nutrient cycling continuously declined in all districts, while the ESVs for hydrology regulation, climate regulation and water resources increased significantly in suburban districts. The ESV imbalance in urban area was more obvious, and the two levels were significant, with “high central area” and “low suburban region”. The high value regions of urban ecosystem services are largely concentrated in water areas. This provides important reference for the policy-making of urban sustainable development.
(3) We confirmed that total fishery output, grain crop output, and total registered residence population are positively associated with ESVs in Wuhan, while negatively correlated with Population density, per capita GDP, and water resources. Human activities play a more important role in urban ecosystem compared to natural environmental factors. Agriculture, GDP, Population and water resources are the dominant factors affecting ESV in urban area. For the population system, the causal relationship between total population and ESV is positively correlated, while the relationship between population density and ESV shows an opposite trend.

This study contributes a new UV method for urban ESV evaluation by improving equivalent factors for built-up land and man-made wetland. Adjustment factors are developed to evaluate joint impacts of natural resources, socioeconomic activities, and spatiotemporal heterogeneity on ESVs. The new UV method supported by these adjustment factors is advantageous over previous UV methods which did not consider spatiotemporal heterogeneity. In addition, by using nine land use and land cover types and establishing a two-level (city and district) ESV assessment system, this study addresses the oversimplification issue rooted in previous UV methods.

Despite the aforementioned contributions are offered in this study, there are still some limitations and directions for future research. Firstly, the data in this research are from 1996 to 2018 due to the availability of data, while there are still shortcomings in reflecting the long-term development trend. Therefore, we need to collect longer-term and more refined artificial, natural, and socio-economic data for ESV evaluation. Secondly, this paper added built-up area and man-made wetland and adjusted the value coefficients of ecosystem services to reduce the uncertainties in urban ecosystem, but neglecting some specific ecosystem services, such as cultural ecosystem services, which are becoming increasingly important in cities and exhibit significant interannual variability. In the future, quantitative assessment method combined field surveys can be used for comprehensive evaluation of cultural ecosystem services. Hence, a unified evaluation method of dynamic equivalent factors for urban ecosystem services requires further exploration in future researches.
